# Efferent Activity Controls Hair Cell Response to Mechanical Overstimulation

**DOI:** 10.1523/ENEURO.0198-22.2022

**Published:** 2022-07-07

**Authors:** Chia-Hsi Jessica Lin, Dolores Bozovic

**Affiliations:** 1 Department of Physics and Astronomy, University of California, Los Angeles, Los Angeles, CA 90095; 2 California NanoSystems Institute, University of California, Los Angeles, Los Angeles, CA 90095

**Keywords:** efferent stimulation, hair bundle mechanics, hair cell, mechanical overstimulation, sacculus

## Abstract

The efferent pathway strengthens the auditory system for optimal performance by fine-tuning the response and protecting the inner ear from noise-induced damage. Although it has been well documented that efference helps defend against hair cell and synaptic extinction, the mechanisms of its otoprotective role have still not been established. Specifically, the effect of efference on an individual hair cell’s recovery from mechanical overstimulation has not been demonstrated. In the current work, we explored the impact of efferent stimulation on this recovery using *in vitro* preparations of hair cells situated in the sacculi of American bullfrogs (*Rana catesbeiana*). In the absence of efferent stimulus, exposure of a hair bundle to high-amplitude mechanical deflection detuned it from its oscillatory regime, with the extent of detuning dependent on the applied signal. Efferent actuation concomitant with the hair bundle’s relaxation from a high-amplitude deflection notably changed the recovery profile and often entirely eliminated the transition to quiescence. Our findings indicate that the efferent system acts as a control mechanism that determines the dynamic regime in which the hair cell is poised.

## Significance Statement

Intense sounds are capable of elevating hearing thresholds and causing permanent hearing loss – a significant and potentially increasing public health problem. The efferent system has been directly implicated in mitigating noise-induced deterioration in the inner ear. However, it is still not known how efferent stimulation performs its protective role at the level of an individual hair cell. In this study, we demonstrate that efferent activation modulates the dynamics of hair bundle recovery following mechanical overstimulation. This finding indicates that the efferent system provides a biological feedback mechanism that controls the dynamic state of a hair cell.

## Introduction

The auditory system is crucial for survival, navigation, and communication among animals of numerous species. In order to perform these functions, the system must be able to detect extremely weak signals and frequently parse these signals from an environment crowded with multiple competing streams of information. To achieve this, the auditory system relies on efferent neurons for control over its sensitivity of detection (Art et al., 1982; Highstein, 1991; Micheyl and Collet, 1996; Micheyl et al., 1997; Darrow et al., 2006; Rabbitt and Brownell, 2011; Smith and Keil, 2015; Guinan, 2018; Lopez-Poveda, 2018; Wang et al., 2021).

Furthermore, in conjunction with its signal detection sensitivity, the delicate machinery of the inner ear must withstand loud sounds without sustaining immediate and irreparable damage. Intense sounds have been observed to cause temporary threshold shifts in humans and other mammals, as well as to have an effect on cochlear microphonics. The transient nature of this noise-induced hearing loss indicates an underlying protective mechanism against permanent damage (Clark, 1991; Melnick, 1991; Patuzzi, 2002; Lichtenhan and Chertoff, 2008; Díaz et al., 2021).

The efferent system has been linked to the auditory system’s inherent otoprotective capacity (Handrock and Zeisberg, 1982; Reiter and Liberman, 1995; Maison and Liberman, 2000; Taranda et al., 2009; Boero et al., 2018; Díaz et al., 2021; Ohata et al., 2021). A number of studies have detected higher levels of noise-induced damage (hair cell and synaptic deterioration) in subjects with severed efferent innervation (Zheng et al., 2000; Maison et al., 2002, 2013; Kujawa and Liberman, 2009; Taranda et al., 2009). As noise-induced hearing loss presents a significant public health problem, it is important to fully understand the role of the efferent system in protecting the auditory epithelia from acoustic trauma.

The hair cell is a fundamental element of the inner ear and derives its name from the organelle, termed the hair bundle, that resides on its apical surface. The hair bundle consists of actin-filled stereovilli arranged in a semi-crystalline pattern, with the mechanically gated ion channels of each row of stereovilli connected to an adjacent row of stereovilli via tip links. When a hair bundle is deflected in the positive direction toward the kinocilium, the hair cell is depolarized by the resulting influx of ions entering the cell through the open transduction channels. This depolarization causes the release of neurotransmitters at the hair cell’s afferent synapse (Hudspeth, 1983).

While little is known about the specific mechanisms by which efference controls the responsiveness of the auditory system, recent observations have demonstrated that activation of efferent neurons strongly modulates the mechanical sensitivity of a hair cell (Lin and Bozovic, 2020). Another set of studies has shown signatures of an internal feedback process, which was observed *in vitro* in isolated sensory epithelia. Specifically, intense mechanical stimulation presented to the hair bundle was shown to induce a transition from the oscillatory state to quiescence (Kao et al., 2013). However, thus far, neither a biological feedback mechanism that controls a hair cell’s response to mechanical overstimulation nor the dynamics of a hair bundle’s subsequent recovery have been identified.

In this work, we investigate the hypothesis that activating the efferent system directly affects a hair cell’s recovery from large mechanical deflections. This hypothesis was tested with experiments conducted on individual hair bundles within semi-intact preparations that preserve the hair bundles’ active motility. We begin by examining the oscillatory characteristics of a hair bundle regaining its innate dynamics after a severe mechanical deflection. Various protocols of efferent actuation are then paired with mechanical overstimulation to probe the impact of efference on a hair cell’s dynamics of recovery.

## Materials and Methods

### Biological preparation

Research was conducted following animal-handling and euthanasia protocols approved by the University of California, Los Angeles Chancellor’s Animals Research Committee, in accordance with federal and state regulations. Experiments were performed on *in vitro* preparations of sacculi extracted from the inner ears of adult North American bullfrogs (*Rana catesbeiana*) of both genders. In order to mimic the physiological conditions of the *in vivo* environment, saccular maculae were affixed in a two-compartment chamber. The basolateral and apical membranes were submerged in artificial perilymph (in mm: 110 Na^+^, 2 K^+^, 1.5 Ca^2+^, 113 Cl^–,^ 3 D-(+)-glucose, 1 Na^+^ pyruvate, 1 creatine, and 5 HEPES) and artificial endolymph (in mm: 2 Na^+^, 118 K^+^, 0.25 Ca^2+^, 118 Cl^–,^ 3 D-(+)-glucose, and 5 HEPES), respectively. Solutions were freshly oxygenated before use. The otolithic membrane was gently lifted from the epithelium subsequent to 9 min of enzymatic dissociation with 50 μg/ml collagenase IA-S (Sigma-Aldrich). After the otolithic membrane was decoupled, spontaneous oscillations were observed in hair bundles and could be sustained for several hours following dissection.

### Imaging and tracking hair bundle motion

Experiments were performed with an upright optical microscope (Olympus BX51WI) mounted on a vibration-isolation table (Technical Manufacturing Corporation) and imaged with a water immersion objective (Olympus XLUMPlanFL N, 20×, 1.00 NA). Images were optically magnified (∼400× total magnification) and recorded with a high-speed CMOS camera (Photron FASTCAM SA1.1) at 1000 frames per second. A hair bundle’s movement was tracked using software written in MATLAB (The MathWorks), and its position was determined by the center of gravity of the bundle’s intensity profile along a row of pixels. This calculation was performed with at least 10 rows of adjacent pixels to enhance the signal-to-noise ratio. The time-dependent position trace of a hair bundle’s motion was acquired by plotting the mean position for each frame of the recording.

### Efferent stimulation

**Figure 1.**
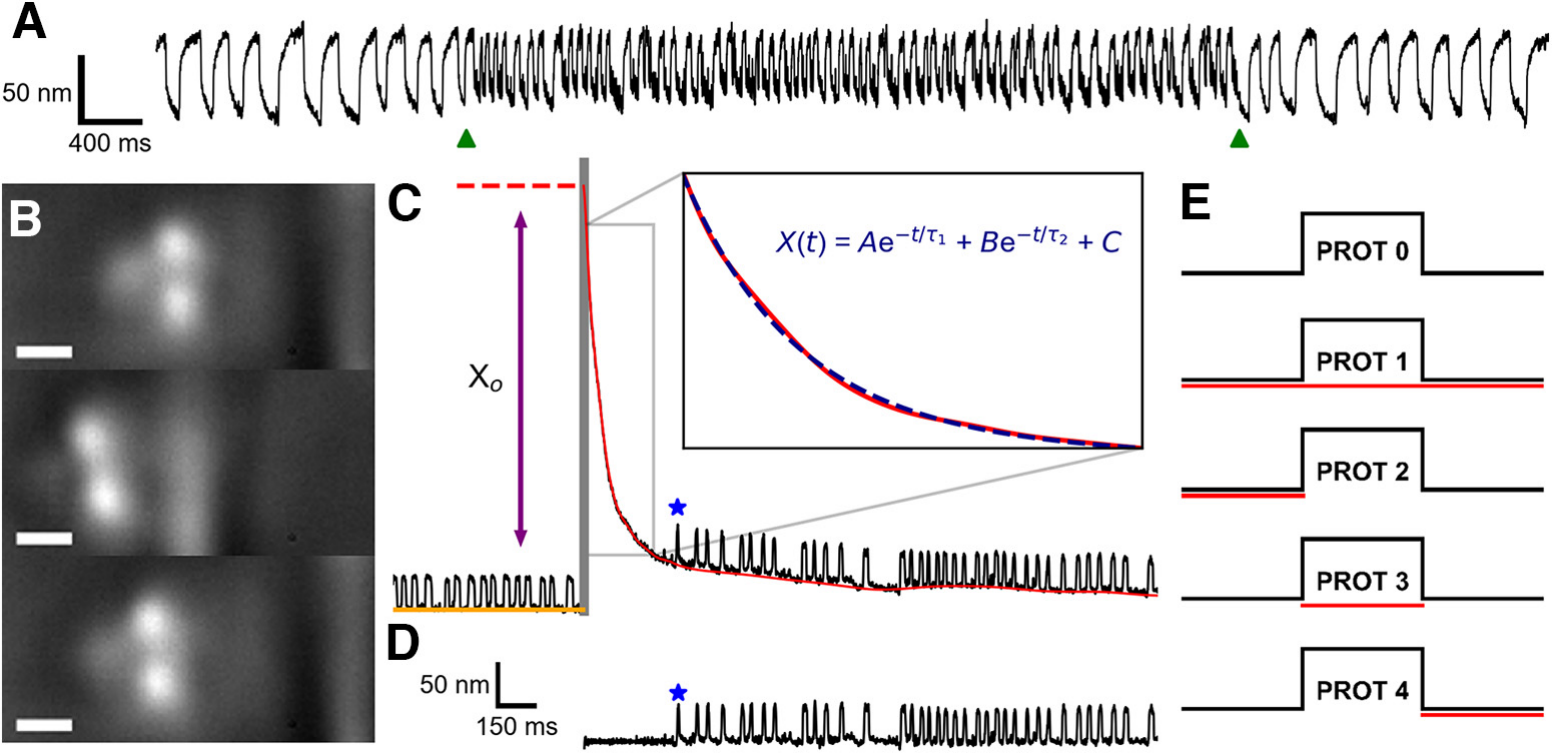
***A***, Spontaneous oscillations before, during, and after efferent stimulation (pulse train: 200 μA, 1 ms on, 10 ms off) are displayed for a representative hair bundle. The onset and offset of the efferent stimulus are indicated by the two green triangles located below the position trace. ***B***, Bright field images showing the application of a large-amplitude (∼1 μm) mechanical deflection to an individual hair bundle via a stiff glass probe. Top, middle, and bottom panels, Hair bundle before, during, and immediately after the mechanical overstimulation, respectively. The width of the scale bar is 1 μm. ***C***, Hair bundle position traces (black) are extracted from high-speed recordings of hair cells undergoing mechanical overstimulation. Hair bundles experience an induced offset before relaxing back to their initial oscillatory dynamic state. The gray vertical bar represents the interval during which the deflection is applied. The baseline (red) of a hair bundle’s relaxation trace (black) is subtracted from the original trace to obtain a flattened recovery trace (***D***). The blue star annotates the location of the first detected oscillation. The initial (induced) offset, *X_o_*, is the height difference between the position of the hair bundle directly after the withdrawal of the glass probe (red dotted line) and the baseline of the prestimulus spontaneous oscillations (orange line). The baselines are fitted to a function (blue dashed line) that consists of the sum of two exponentials with time constants *τ*_1_ and *τ*_2_ (where $\tau_{2}>\tau_{1}$). Scale bars in ***D*** are applicable for ***C***. ***E***, The five stimulation protocols were designed as follows: protocol 0 had no efferent stimulation and solely consisted of a large mechanical deflection (black line). Protocol 1 included 20 s of efference (200 μA, 3 ms on/10 ms off) before, during, and after the mechanical overstimulation (indicated by the red line). Protocol 2, protocol 3, and protocol 4 featured efferent actuation exclusively before, during, or after the mechanical overstimulation, respectively.

The saccular nerve was stimulated using a bipolar suction electrode (AM Systems; Lin and Bozovic, 2020) and electrically connected to the positive electrode via a 0.5 mm diameter silicon tube (Castellano-Muñoz et al., 2010). The reference electrode was positioned in the basolateral compartment. Current was supplied to the suction electrode via a linear stimulus isolator (World Precision Instruments A395), and stimulus protocols were sent to the isolator via LabView (National Instruments). On its own, efferent modulation immediately altered the oscillation profile of a spontaneously oscillating hair bundle (Fig. 1*A*). Throughout this work, efferent modulation was delivered in the form of a 200 μA pulse train with a 3 ms pulse duration and a 10 ms interpulse interval. In experiments featuring dual mechanical and electrical stimulation of the hair cell, a five protocol efference paradigm was used (Fig. 1*E*). The efferents were not actuated in protocol 0, and thus protocol 0 was treated as the control against which comparisons were made. In protocol 1, the efferent neurons were activated before, during, and after the standard mechanical overstimulation for a total of 60 s. Protocol 2, protocol 3, and protocol 4 present efferent modulation exclusively before, during, and after the mechanical overstimulation, respectively.

### Mechanical overstimulation

Prolonged mechanical overstimulation in the positive direction (toward the kinocilium) was applied with a stiff glass probe ∼1–2 μm in diameter (Fig. 1*B*). Glass fibers were initially pulled from borosilicate glass capillaries in a micropipette puller (Sutter Instruments P-97). Then, an additional rod was fabricated perpendicular to the tip of the pulled capillary with a modified microforge. In order to depress the natural adhesion that occurs between a hair bundle and a glass fiber, the fiber tips were coated with a layer of hydrophobic silane (Gelest SII6453.0). This ensured that a hair bundle’s recovery was not impacted by artifacts introduced from adhesion to the glass probe. Probes were mounted on a piezoelectric actuator (Piezosystem Jena PA 4/12) and positioned ∼2 μm away from the tallest row of stereocilia. Step signals corresponding to an ∼1 μm deflection were generated and digitally filtered (eight-pole Bessel filter at 300 Hz) in Python. Before experimentation, visual confirmation ensured that the glass fiber deflected the hair bundle by ∼1 μm. Step deflections were sent to the piezoelectric amplifier concurrently with video acquisition via LabView (National Instruments). In order to account for the glass probe’s hydrodynamic effects in our analysis, surveillance of the bundle’s recovery response initiated only once the probe was fully withdrawn. Large-scale displacements of the hair bundle (in the direction of the kinocilium) provided mechanical overstimulation that detuned the hair cells away from their natural dynamic state (Kao et al., 2013). Prior work indicated that a sustained steady-state deflection elicited comparable effects as prolonged high-amplitude sinusoidal deflection (Shlomovitz et al., 2013). Thus, the prolonged static stimulus of the hair cell was employed as a simplified model of the hair cell’s response to loud sound stimuli.

### Data analysis

#### Baseline extraction from overstimulation recovery

Recovery of active bundle motility following mechanical overstimulation involves both a return to the bundle’s initial equilibrium position and a reemergence of bundle oscillations (Kao et al., 2013). To disentangle these two components from the original recovery trace, we calculated the baseline trend in two steps. First, a rough estimation of the baseline was computed by passing the original recovery trace through a moving average filter (5 points window) five times, and the time at which the slope of the rough baseline estimation surpassed −0.02 nm/ms was determined. Then, a narrow moving average filter (11 points window) was implemented three times for position values before this threshold time, and a broader average filter (1000 points window) was applied three times for the subsequent data points. The final baseline trend, superposed on the original trace, shows the results of the extraction procedure (Fig. 1*C*). Isolating the slow component of the recovery and subtracting it from the original recording resulted in a “flattened” recovery trace (Fig. 1*D*), which allowed for ready visualization of the oscillation onset. We note that this approach yielded similar results to the two-exponent fit used previously (Kao et al., 2013).

#### Exponential fits of recovery baselines

In order to analyze the slow component of a hair bundle’s recovery, we calculated the characteristic time constants (*τ*_1_ and *τ*_2_, where we define $\tau_{2}>\tau_{1}$) of the extracted baselines by fitting them to the following function:

(1) $$X(t)=A\exp(-\frac{t}{\tau_{1}})+B\exp(-\frac{t}{\tau_{2}})+C,$$

where *A*, *B*, and *C* are multiplicative constants (Fig. 1*C*).

### Determination of instantaneous oscillation frequency, amplitude, and open probability

Throughout this manuscript, a positive displacement in a trace corresponds to motion toward the kinocilium, which is consistent with the standard convention in the literature. Our analysis included only those position traces (flattened or otherwise) for which the hair bundle’s activity was classified as oscillatory by satisfying the criteria of multimodality in the position distribution (Hartigan and Hartigan, 1985; Salvi et al., 2015, 2016; Lin and Bozovic, 2020). For all position traces, the open-channel threshold was computed by first calculating the histogram of the bundle position and applying a kernel density estimate to obtain a continuous distribution. The open-channel threshold was then defined as the minimum position between the two peaks. The instantaneous frequency was acquired by inverting the period between two consecutive, positive transitions from the closed-channel state to the open-channel state. For the composite data points of a single cycle, those points above and below the threshold were separately averaged to give a top and bottom mean. The amplitude of each cycle was defined to be half of the difference between the two means. The mean open probability for each cycle was inferred by dividing the amount of position data points with values exceeding the threshold by the total number of data points in an individual cycle. This estimate was based on electrophysiological measurements, which demonstrated that a hair cell’s mechanoelectrical transduction (MET) current closely correlates with the bundle position over the course of its oscillation (Meenderink et al., 2015). Hence, the fraction of the oscillation cycle corresponding to the positive deflection of the bundle provides a close estimate for the mean open probability of the MET channel during that cycle. Instantaneous parameters tabulated for an analyzed trace were averaged to obtain the mean oscillation parameters. Trendlines were generated by way of a moving average filter.

### Determination of quiescent time and initial offset

Two additional parameters, quiescent time and initial offset, were extracted from the overstimulation recovery position traces. The quiescent time, *T_q_*, is the length of time between the cessation of the overstimulation and the first oscillation, as determined by the process detailed in the previous section. The initial offset, *X_o_*, was defined as the height difference between the apex of the original recovery trace and the baseline of the spontaneous oscillations before mechanical overstimulation (Fig. 1*C*).

### Statistical analysis

Assessments of the statistical significance of the measured difference between a condition and the control for a population were performed using a one-tailed paired *t* test. Differences were considered significant if *p *<* *0.05. The sample size was deemed sufficient if the resultant *p* values met the requirement for statistical significance. As this work is focused on probing individual hair cells, the sample size was defined as the number of hair cells observed.

## Results

The current study investigates the influence of efferent modulation on a hair bundle’s active recovery dynamics following mechanical overstimulation. We started with an examination of the temporal evolution of a hair bundle’s oscillation profile after it has experienced a large mechanical deflection. Subsequently, we explored the effects of incorporating efferent activity with the mechanical stimulus.

### Hair bundles express gradual recuperation following mechanical overstimulation

**Figure 2.**
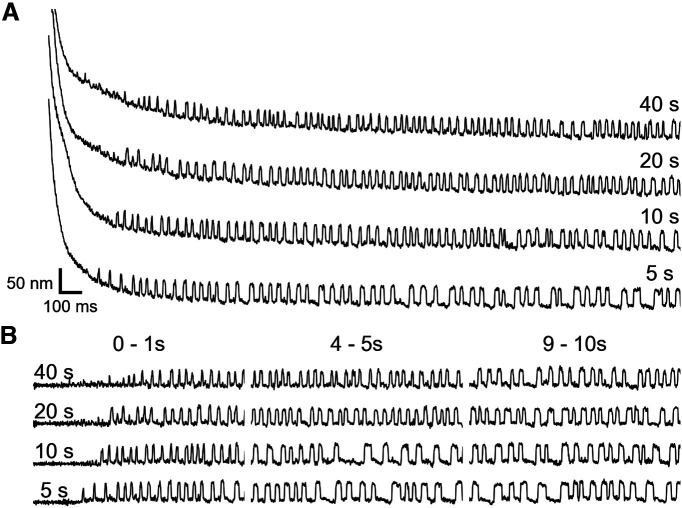
A hair bundle’s relaxation trajectory is dependent on the duration of mechanical overstimulation. ***A***, A series of traces recorded from a single hair cell is shown. Each trace depicts hair bundle recovery following mechanical overstimulation of duration (in seconds) indicated on the right. The hair bundle remains in a quiescent state longer with increasing stimulus duration. The recording order was from bottom to top. ***B***, A series of flattened recovery traces, extracted from the recordings displayed in ***A***, is shown. Three, chronologically subsequent segments are displayed in the first (0–1 s), second (4–5 s), and third (9–10 s) panels. Longer ODs lead to slower recovery of the original oscillation profile. Scale bars in ***A*** are applicable for ***B***.

Hair bundles of the amphibian sacculus have been shown to exhibit spontaneous oscillations, active motion observed in the absence of stimulus, which are indicative of an interplay between mechanical transduction and internal adaptation processes (Martin et al., 2003; Maoiléidigh and Ricci, 2019). This active motility is sensitive to changes in Ca^2+^ concentration, membrane potential of the soma, and other manipulations, and thus provides us with a useful experimental readout of the dynamic state of the hair bundle (Bozovic and Hudspeth, 2003; Martin et al., 2003; Le Goff et al., 2005; Ramunno-Johnson et al., 2010; Roongthumskul et al., 2011; Meenderink et al., 2015; Salvi et al., 2015; Lin and Bozovic, 2020). In this subsection, we report on the findings of experiments on saccular hair cells solely experiencing mechanical overstimulation, and examine its impact on the innate oscillations of the bundle. We applied large (∼1 μm) mechanical deflections to hair bundles via a silane-coated, stiff glass probe. The deflections were applied for an overstimulation duration (OD) of 5, 10, 20, or 40 s. Bundles were deflected in the positive direction (toward the kinocilium), which corresponds with opening transduction channels. Recordings began 2 s before the application of the stimulus and concluded 20 s after the probe was retracted. All of the examined hair cells exhibited robust spontaneous bundle oscillations before mechanical overstimulation. Figure 2*A* portrays a hair bundle’s time courses of recovery following each of the four ODs. We observed both a transitory suppression of spontaneous oscillations and a temporary offset in bundle position, consistent with observations by Kao et al. (2013).

**Figure 3.**
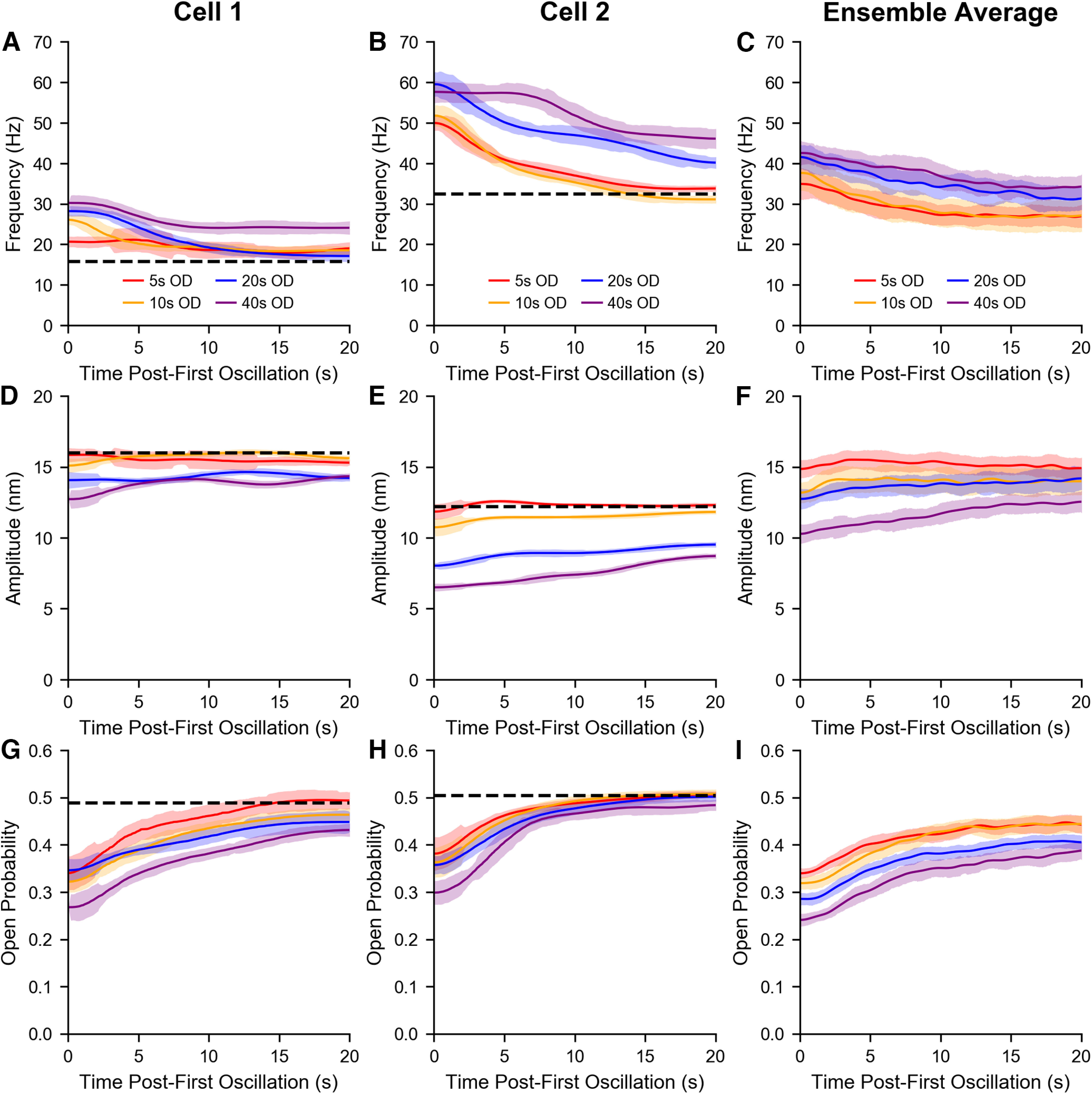
Varying the duration of hair bundle deflection affects the induced shifts in the oscillation parameters. Time-series trendlines of instantaneous frequencies, amplitudes, and inferred MET channel open probabilities are plotted for the hair cell in Figure 2 (***A***, ***D***, ***G***) and another cell from a different sacculus (***B***, ***E***, ***H***). The four ODs (5, 10, 20, 40 s) are plotted in red, gold, blue, and violet, respectively. The black dashed lines indicate the corresponding values of the cell’s original dynamic state. A hair bundle re-enters the oscillatory regime displaying different characteristics from its initial state. The oscillation parameters reflect this difference before gradually transitioning back to their characteristic values. Frequency, amplitude, and open probability trendlines of the same OD were averaged together to obtain the mean trendlines in ***C***, ***F***, ***I***, respectively. As the OD increases, the initial frequency increases, while the amplitude and open probability decrease. This increased detuning from the original state correlates with a slower recovery from longer mechanical overstimulation. The averaged trendlines reflect data from 13 bundles (6 sacculi). Error bands represent the SDs of data points in a 1 s moving window.

To quantify the effects of mechanical deflections on the temporal profiles of hair bundle oscillations, we examined the trendlines of the instantaneous frequencies, amplitudes, and open probabilities of flattened traces and investigated the time dependence of recovery to the original oscillatory state. The trendlines presented in Figure 3*A*,*D*,*G* and *B*,*E*,*H* were calculated from the traces in Figure 2*B* and a cell selected from a different sacculus, respectively. Frequency, amplitude, and inferred MET channel open probability trendlines extracted from 13 bundles (6 sacculi) were averaged, and the mean trendlines for each OD are depicted in Figure 3*C* (frequency), *F* (amplitude), and *I* (open probability). Figure 3*C* indicates that the initial frequency of spontaneous oscillation for a hair cell recovering from quiescence is dependent on the OD, longer ODs gave rise to higher initial frequencies. In all cases, the frequency trendlines progressed toward a plateau. Figure 3*F* shows the pattern obtained for amplitude recovery, longer ODs led to smaller initial amplitudes. The trendlines of the four conditions gradually regressed toward a common point. Finally, Figure 3*I* shows a negative correlation between a bundle’s nascent open probability and its OD. Similar to Figure 3*F*, the four mean trendlines arch upwards until leveling off. The conclusions drawn from the mean plots can also be inferred from the individual bundle plots. Figure 3 indicates that longer ODs shift a hair cell further away from its original dynamic state.

### Efferent modulation influences hair cell recovery from mechanical overstimulation

**Figure 4.**
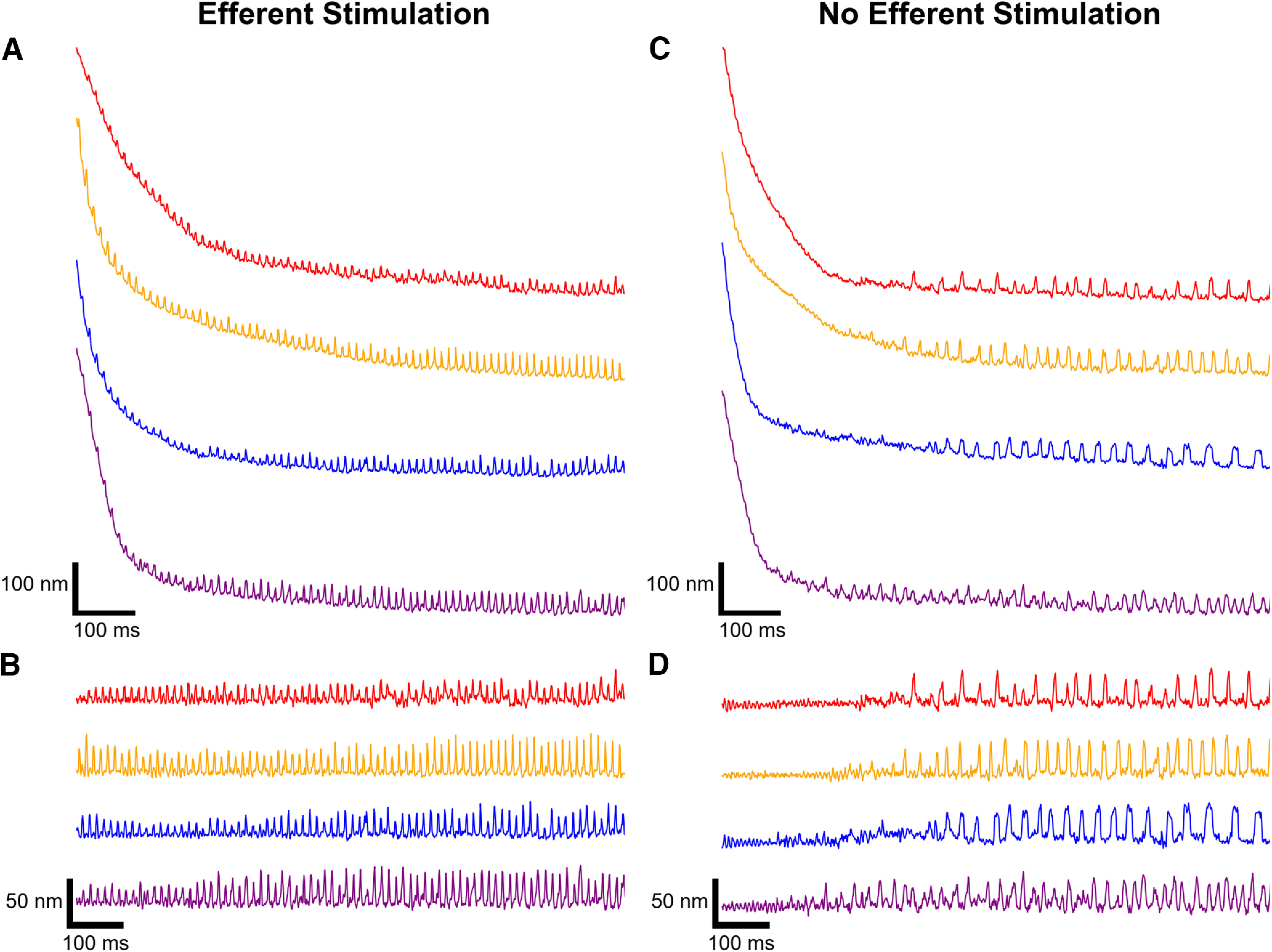
Stimulation of efferent neurons provokes an immediate crossover from the quiescent state back to the oscillatory regime. Position traces of four example hair cells recovering from mechanical overstimulation (20 s OD) with concurrent efferent actuation (***A***) illustrate hair bundles in an oscillatory state despite the large positional offsets. Analogous recordings obtained without concurrent efferent actuation (***C***) show an initial quiescent interval. ***B***, ***D***, Flattening the traces in ***A*** and ***C***, respectively, confirms that a high-amplitude mechanical deflection does not halt oscillatory motion when the efferents are simultaneously activated. Each of the four bundles originated from a distinct sacculus, shown in different colors and offset for clarity. All traces corresponding to the same hair cell are displayed in the same color.

Subsequently, we explored the impact of efferent modulation on a hair cell’s recovery from mechanical overstimulation. For these experiments, the OD was kept constant at 20 s, and we recorded hair bundle recovery both in the presence and absence of efferent stimulation. When efferent activation was concomitantly in effect with a hair bundle’s overstimulation recovery, we routinely observed bundles whose oscillatory motion was not abated by the 20 s large-amplitude mechanical deflection (Fig. 4*A*). By contrast, all such recordings obtained without efferent stimulation (Fig. 4*C*) showed the usual suppression of bundle motility. Under both stimulus paradigms, the bundles showed a significant and comparable accumulated offset in their position, an effect discussed in a subsequent section. Efferent actuation seemed to annul the effect of the induced offset and allow for oscillations to return even during the steep initial portion of the recovery from deflection.

For clarity, we also show flattened traces for this set of recordings (Fig. 4*B*), which demonstrate that stimulating the efferent neurons was able to provoke an immediate crossover from the quiescent state to the oscillatory state before recovery from the accrued offset. The corresponding flattened traces obtained in the absence of efferent activity (Fig. 4*D*) display an initial suppression of active motility. We note that the display of this immediate oscillatory behavior varied greatly between individual hair bundles, with a fraction of observed hair cells (19 of 46 cells recorded across 16 preparations) expressing this feature.

This surprising finding indicates that a high-amplitude mechanical deflection does not necessarily halt innate oscillatory motion when the efferents are simultaneously activated. In the subsequent sections, we analyze the temporal dynamics of this influence, the disparate effect on the induced positional offset and oscillatory motion, and variability among bundles.

### Parsing the temporal dependencies of efferent modulation

To parse the impact of efferent activity on different intervals of hair bundle deflection and recovery, we examined the response to overstimulation under five different efferent stimulus paradigms. The protocols were designed without any a priori assumptions as to which component of the stimulation or recovery interval is susceptible to efferent control. In all cases, the duration of mechanical deflection was kept constant at 20 s. The five stimulation protocols were designed as follows (Fig. 1*E*): protocol 0 had no efferent stimulation and solely consisted of a large mechanical deflection, thus constituting the control against which comparisons were made. Protocol 1 included 20 s of efference (200 μA, 3 ms on/10 ms off) before, during, and after the mechanical overstimulation. Protocol 2, protocol 3, and protocol 4 featured efferent actuation exclusively before, during, or after the mechanical overstimulation, respectively. All recordings began 20 s before the onset of the probe deflection and concluded 20 s following the probe’s retraction for a total recording time of 60 s. All reported hair bundles displayed robust spontaneous bundle oscillations before mechanical overstimulation.

**Figure 5.**
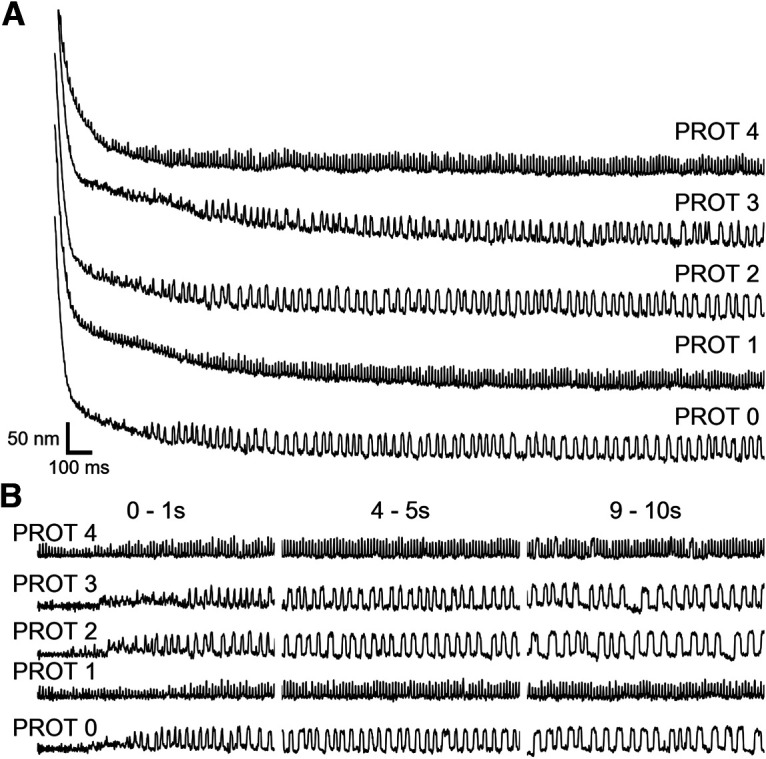
Different intervals of efferent stimulation distinctly affect a hair bundle’s oscillation profile as it recovers from mechanical overstimulation. ***A***, A series of traces are shown of recordings from a hair cell undergoing a combination of mechanical overstimulation and efferent actuation. Each trace depicts hair bundle motion following 20 s of large-amplitude mechanical deflection combined with the efference paradigm indicated on the right. The efferents are not actuated in protocol 0, and thus protocol 0 is treated as the control condition against which comparisons are made. In protocol 1, the efferent neurons are activated before, during, and after the mechanical overstimulation for a total of 60 s. Protocol 2, protocol 3, and protocol 4 present efferent modulation exclusively before, during, or after the mechanical overstimulation, respectively. The recording order is from bottom to top. A portion of the observed hair bundles exhibit oscillatory motion immediately on probe release. ***B***, A series of flattened recovery traces corresponding to the traces in ***A*** is shown. Three, chronologically subsequent segments are displayed in the first (0–1 s), second (4–5 s), and third (9–10 s) panels. When efference is present during the hair bundle’s recovery (protocol 1 and protocol 4), the bundle’s oscillation profile is significantly altered. Scale bars in ***A*** are also applicable for ***B***.

Figure 5*A* illustrates an individual hair bundle’s recovery trajectories for each of the five efference paradigms. We performed the full set of experimental protocols on 18 hair cells across five sacculi. In Figure 5*B*, we plot the flattened traces, with the first column showing the initial recovery of oscillation, and the remaining two showing the influence of continued efferent actuation (protocol 1 and protocol 4) on spontaneous oscillation profiles. The trends observed in our recordings indicate that stimulation of efferent neurons either before or during the mechanical stimulus (protocol 2 and protocol 3) did not have a statistically measurable effect on subsequent recovery profiles. Hence, there was no accumulated persistent change in the bundle dynamics that would affect its response. Finally, the protocol 4 paradigm, discussed previously, eliminated the initial suppression of oscillation, showing that concurrent efferent activity strongly changes the dynamics of recovery. The protocol 1 paradigm, which included all intervals of the efferent stimulus, was statistically indistinguishable from protocol 4.

### Efference strongly impacts the recovery of active oscillations

**Figure 6.**
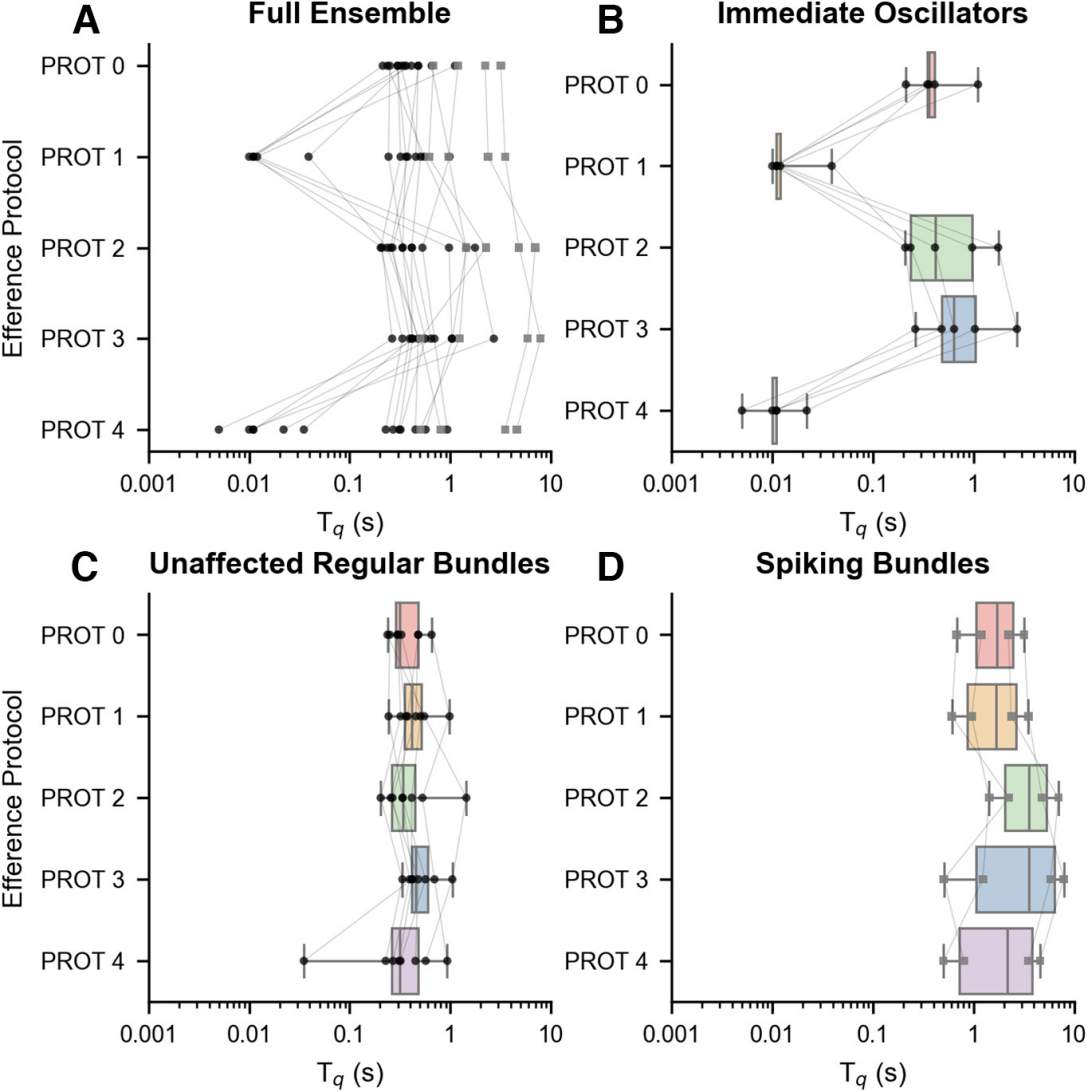
Efferent modulation exerts an effect on the quiescent interval observed before recovery of active oscillations. ***A***, A distribution of quiescent times (*T_q_*) across the five efference paradigms, obtained from recordings of 18 bundles across five sacculi, is shown. Bundles whose spontaneous oscillations exhibited “spiking” behavior generally had longer quiescent times and are specifically marked with gray squares. Data points from the same hair bundle are connected together. Efference paradigms that feature efferent activation during the postoverstimulation period (protocol 1 and protocol 4) displayed wider ranges of quiescent times, with seven bundles having their first oscillation occurring within 50 ms. The recordings were separated into three groups: hair bundles that display an immediate return to oscillation (***B***), those that display regular oscillations and are not immediately affected by efference (***C***), and bundles that exhibit spike-like motion (***D***). Box plots illustrate the distribution of quiescent times observed in each group.

We subsequently investigate how the full set of efference paradigms affected different components of the recovery. First, we examine in detail the quiescent times, intervals during which innate oscillations are suppressed, extracted from the recordings obtained under different efference protocols (Fig. 6*A*). Data points originating from the same bundle are linked together. Since we observed that hair bundles whose spontaneous motility exhibited a “spiking” profile (sharp, brief excursions from the channel-closed state, correlating with an average inferred MET channel open probability <0.2) generally displayed longer quiescent periods, these bundles are distinguished from the others by the use of gray square markers.

Efference paradigms that feature efferent activation during the recovery period (protocol 1 and protocol 4) demonstrated a wider range of quiescent times. A clear dichotomy emerged in the observed behavior: a subset of bundles resumed oscillations immediately on cessation of the stimulus, and a subset whose quiescent times showed little or no effect of efferent modulation. We grouped hair bundles into separate subsets to analyze them independently and to determine whether the elimination of quiescence correlated with other features in the recovery dynamics.

In the first subset (Fig. 6*B*), we grouped together bundles that displayed an immediate return to oscillation. A hair cell was classified as immediately oscillatory if both the protocol 1 and protocol 4 quiescent times were <50 ms. The second and third subsets contain bundles that were not immediately affected by efference; these in turn are grouped based on whether they initially exhibited regular (Fig. 6*C*) or spiking (Fig. 6*D*) oscillations. Each subset was analyzed separately, and the quiescent time (*T_q_*) averages of each efference protocol for the immediate oscillators (Fig. 6*B*) are listed in the $〈T_{q}〉$ column of Table 1. With respect to protocol 0, the differences in quiescent times and the results of one-tailed paired *t* tests are shown in Table 1.

**Table 1.** Average quiescent times measured under different efference protocols

		〈*T_q_*〉	Δ w.r.t. PROT 0	One-tailed paired *t* test	
				*t* statistic	*p* value
Immediate oscillators	PROT 0	0.49 ± 0.32 s	—	—	—
	PROT 1	0.02 ± 0.01 s	−0.47 ± 0.32 s	*t*_(4)_ = −3.31	*p *=* *0.02*
	PROT 2	0.72 ± 0.59 s	0.23 ± 0.34 s	*t*_(4)_ = 1.53	*p *=* *0.10
	PROT 3	1.03 ± 0.88 s	0.54 ± 0.57 s	*t*_(4)_ = 2.09	*p *=* *0.05
	PROT 4	0.01 ± 0.01 s	−0.48 ± 0.32 s	*t*_(4)_ = −3.35	*p *=* *0.01*
Unaffected regular	PROT 0	0.38 ± 0.14 s	—	—	—
	PROT 1	0.47 ± 0.21 s	0.10 ± 0.15 s	*t*_(8)_ = 1.81	*p *=* *0.06
	PROT 2	0.47 ± 0.38 s	0.09 ± 0.44 s	*t*_(8)_ = 0.62	*p *=* *0.28
	PROT 3	0.54 ± 0.22 s	0.17 ± 0.27 s	*t*_(8)_ = 1.77	*p *=* *0.06
	PROT 4	0.39 ± 0.25 s	0.01 ± 0.20 s	*t*_(8)_ = 0.16	*p *=* *0.44
Spiking	PROT 0	1.82 ± 0.96 s	—	—	—
	PROT 1	1.86 ± 1.16 s	0.04 ± 0.22 s	*t*_(3)_ = 0.41	*p *=* *0.35
	PROT 2	3.87 ± 2.18 s	2.05 ± 1.65 s	*t*_(3)_ = 2.48	*p *=* *0.05
	PROT 3	3.87 ± 3.10 s	2.05 ± 2.16 s	*t*_(3)_ = 1.91	*p *=* *0.08
	PROT 4	2.35 ± 1.74 s	0.53 ± 0.87 s	*t*_(3)_ = 1.23	*p *=* *0.15

Each subset of hair bundle response to simultaneous efferent actuation and mechanical overstimulation was separately analyzed (Fig. 6*B*–*D*), and the quiescent time (*T_q_*) averages of each efference protocol are listed in the $〈T_{q}〉$ column. With respect to protocol 0, the differences in quiescent times and the results of one-tailed paired *t* tests are shown. Differences were considered significant if *p *<* *0.05 and are indicated by asterisks. Thus, protocol 1 and protocol 4 both had statistically significant differences in their mean quiescent times for those hair bundles that exhibited oscillatory motion immediately postoverstimulation. On the other hand, neither the unaffected regular hair cells nor the spiking bundles displayed statistically significant differences in their mean quiescent times with respect to protocol 0 for any of the efference paradigms.

We observed that the subset of hair cells that were collected together based on statistically significant differences in protocol 1 and protocol 4 did not display a statistically significant effect under the other stimulus protocols (protocol 2 and protocol 3). These findings indicate that efferent actuation was primarily effective in eliminating quiescence when applied during the recovery period (after the cessation of the mechanical deflection). Likewise, neither the unaffected regular hair cells nor the spiking bundles presented statistically significant differences in their mean quiescent times under any of the other efference paradigms with respect to protocol 0. The *T_q_* averages for the unaffected regular bundles (Fig. 6*C*) and the spiking subset (Fig. 6*D*) are displayed in the $〈T_{q}〉$ column of Table 1. With respect to protocol 0, the differences in quiescent times and the results of one-tailed paired *t* tests are listed in Table 1.

**Figure 7.**
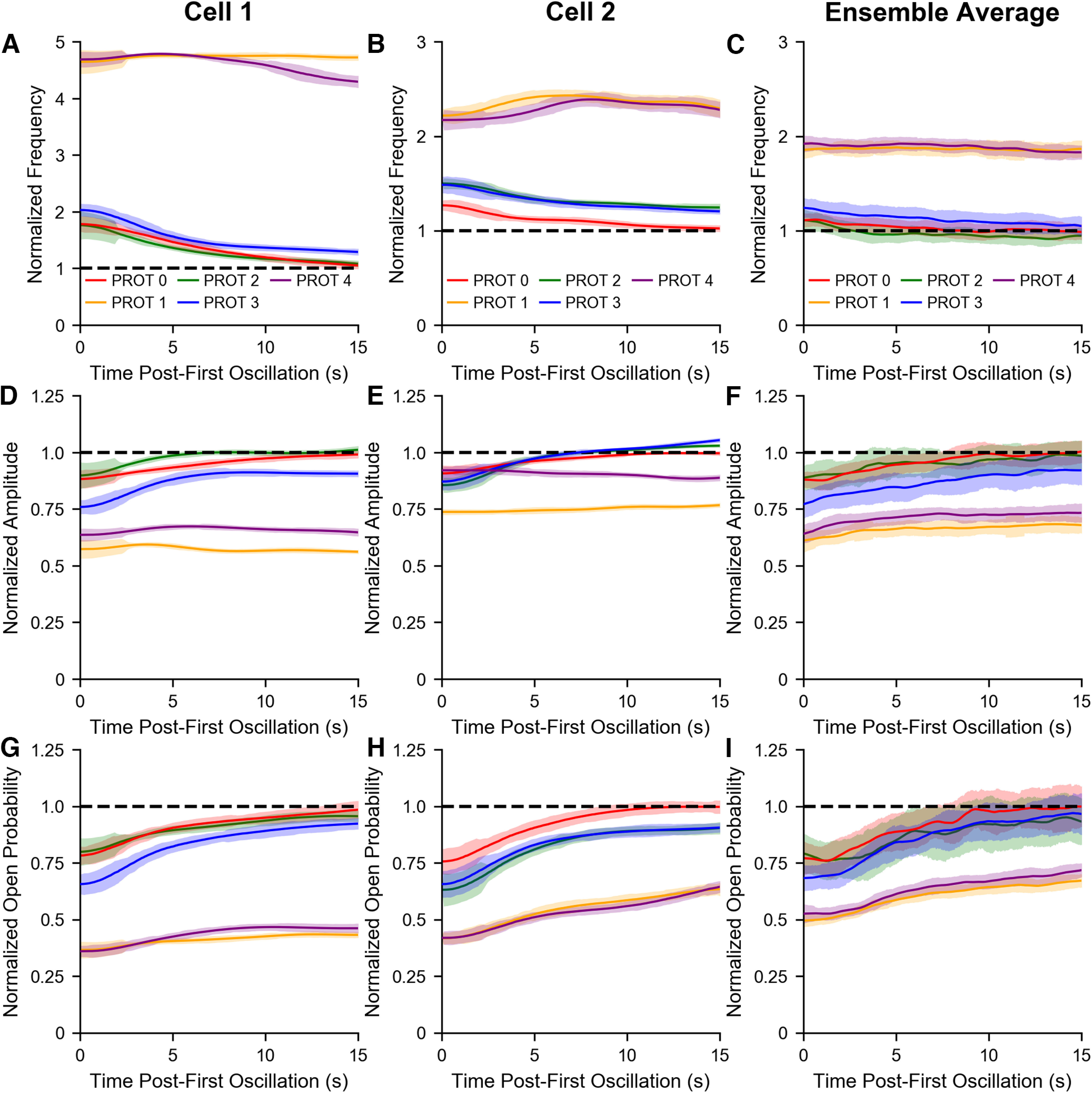
Among the five efference paradigms, a clear difference can be observed between protocols with or without efferent activation during the hair bundle’s recovery. Time-series trendlines of instantaneous frequencies, amplitudes, and inferred MET channel open probabilities are plotted for the hair cell in Figure 5 (***A***, ***D***, ***G***) and another cell from a different sacculus (***B***, ***E***, ***H***). All trendlines were normalized by the specific bundle’s steady state value. The five efference paradigms (protocols 0, 1, 2, 3, 4) are plotted in red, gold, green, blue, and violet, respectively. Under protocol 1 and protocol 4, hair bundles return to the oscillatory regime with a higher frequency, lower amplitude, and smaller open probability than those in the three other protocols. Frequency, amplitude, and open probability trendlines with the same efference protocol were averaged together to obtain the mean normalized trendlines in ***C***, ***F***, ***I***, respectively. The protocol 1 and protocol 4 mean open probability trendlines are initially shifted downwards with respect to the protocol 0 trendline (***I***), but proceed to gradually increase over time, in contrast to the relatively flat frequency (***C***) and amplitude (***F***) trendlines. The averaged trendlines reflect data from 18 bundles (5 sacculi). Error bands represent the SDs of data points in a 1 s moving window.

In addition to the duration of the quiescent interval, we also examined the temporal profiles of the recovering oscillations. We investigated the time-series trendlines of the instantaneous frequencies, amplitudes, and open probabilities of flattened recovery traces for the five efference paradigms. The normalized trendlines displayed in Figure 7*A*,*D*,*G* and *B*,*E*,*H* were calculated from the flattened traces in Figure 5*B* and those of a separate cell, respectively. Normalization was obtained by taking the ratio of the instantaneous values to their respective characteristic values. The individual normalized trendlines from the previous 18 hair bundles were averaged together, and the mean normalized trendlines for each of the five protocols are shown in Figure 7*C* (frequency), *F* (amplitude), and *I* (open probability).

The overall shape and trajectory of the trendlines in Figure 7*C*,*F*,*I* are similar to those seen in Figure 3*C*,*F*,*I*, respectively. However, there is an explicit difference between the efference protocols that do and do not have efferent stimulation during the postoverstimulation recovery, with the respective trendlines generally overlapping. As expected, protocol 1 and protocol 4 frequency trendlines were shifted upwards with respect to the protocol 0 frequency trendline and generally maintained their nascent frequencies for the entire 20 s (Fig. 7*C*). In conjunction, the amplitude trendlines of protocol 1 and protocol 4 were shifted downwards with respect to the protocol 0 amplitude trendline and did not experience the same amplitude rise as those of the three other protocols (Fig. 7*F*), because of the efferent stimulus’s continued effect on the spontaneous oscillations. However, the open probability trendlines of protocol 1 and protocol 4, initially below that of protocol 0, proceeded to gradually increase over time in parallel with the protocol 0 trendline. In total, Figure 7 reveals that the concurrent presence of efferent modulation during the postoverstimulation recovery plays a significant role influencing the attributable characteristics of a hair bundle’s oscillation profile during its recovery from mechanical overstimulation.

### Efferent actuation does not affect the slow dynamics of recovery

**Figure 8.**
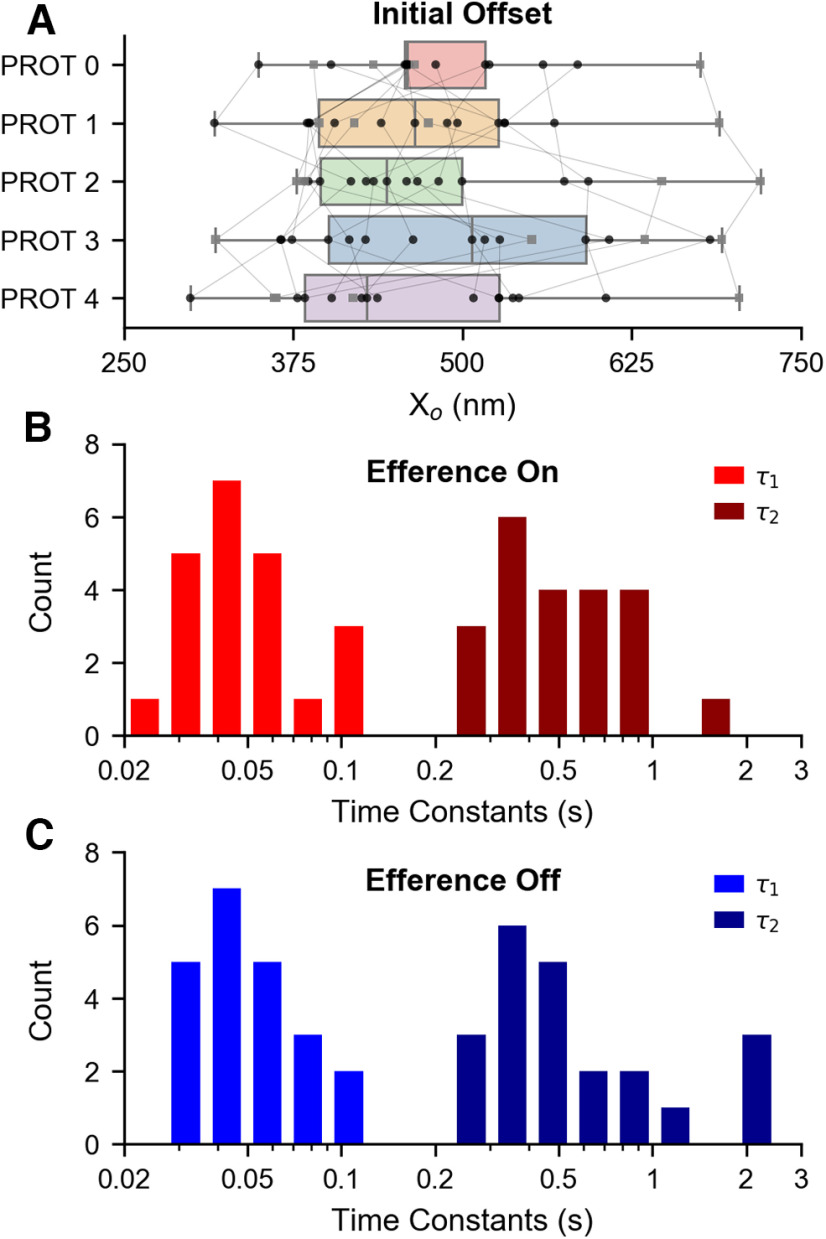
Actuation of the efferent neurons does not significantly affect the slow-component of a hair bundle’s recovery from mechanical overstimulation. ***A***, Box plots illustrate the distribution of initial offsets (*X_o_*) across the five efference paradigms. Bundles whose spontaneous oscillations exhibited “spiking” behavior are specifically marked with gray squares. Data points from the same hair bundle are connected together. There were no statistically significant differences in the mean initial offsets of the four protocols compared with the protocol 0 control. Thus, efferent modulation does not influence a hair bundle’s induced initial offset. Data points in ***A*** were obtained from recordings of 18 bundles across five sacculi. An extracted baseline was fitted to the sum of two exponentials, which yielded two time constants, *τ*_1_ and *τ*_2_ ( $\tau_{2}>\tau_{1}$). The computed baseline time constants for recovery with simultaneous efferent actuation (“efference on”) are shown in ***B***, and the time constants for the same hair bundles obtained without efferent actuation (“efference off” condition) are illustrated in ***C***. The data shown in ***B*** and ***C*** were obtained from recordings of 22 hair bundles extracted from nine sacculi. All baselines were uniquely fitted with *R*^2^ > 0.95. On average, the presence of efferent stimulation does not substantially alter the hair bundle’s resumption of its steady state position.

Subsequently, we examined the effects of the different efference protocols on the accumulated mechanical offset, which was measured immediately on retraction of the probe. The *X_o_* averages (Fig. 8*A*) are as follows: 478.42 ± 74.58, 465.13 ± 86.39, 476.07 ± 97.99, 496.7 ± 113.73, and 461.82 ± 100.03 nm for protocols 0, 1, 2, 3, and 4, respectively. Hence, the average initial offsets postmechanical overstimulation were comparable for all of the efference stimulus paradigms. The hair bundles were subdivided and further analyzed in the same manner as in the prior section, with the mean initial offsets of the three subcategories shown in the $〈X_{o}〉$ column of Table 2. With respect to protocol 0, the differences in initial offsets and the results of one-tailed paired *t* tests are listed in Table 2. Thus, there were no statistically significant differences in the initial offsets for any of the subgroups of hair cells under any of the efference paradigms.

**Table 2.** Average initial offsets measured under different efference protocols

		〈*X_o_*〉	Δ w.r.t. PROT 0	One-tailed paired *t* test	
				*t* statistic	*p* value
Immediate oscillators	PROT 0	495.16 ± 39.30 nm	—	—	—
	PROT 1	493.41 ± 59.89 nm	−1.75 ± 46.53 nm	*t*_(4)_ = −0.08	*p *=* *0.47
	PROT 2	476.20 ± 62.06 nm	−18.96 ± 27.12 nm	*t*_(4)_ = −1.56	*p *=* *0.10
	PROT 3	496.99 ± 111.24 nm	1.83 ± 83.30 nm	*t*_(4)_ = 0.05	*p *=* *0.48
	PROT 4	486.40 ± 93.85 nm	−8.76 ± 80.25 nm	*t*_(4)_ = −0.24	*p *=* *0.41
Unaffected regular	PROT 0	461.59 ± 65.51 nm	—	—	—
	PROT 1	432.71 ± 69.80 nm	−28.88 ± 68.36 nm	*t*_(8)_ = −1.19	*p *=* *0.14
	PROT 2	448.08 ± 61.84 nm	−13.51 ± 61.35 nm	*t*_(8)_ = −0.62	*p *=* *0.28
	PROT 3	470.51 ± 87.07 nm	8.92 ± 89.07 nm	*t*_(8)_ = 0.28	*p *=* *0.39
	PROT 4	446.46 ± 71.17 nm	−15.13 ± 42.97 nm	*t*_(8)_ = −1.00	*p *=* *0.18
Spiking	PROT 0	491.16 ± 109.77 nm	—	—	—
	PROT 1	494.64 ± 116.28 nm	3.47 ± 30.75 nm	*t*_(3)_ = 0.23	*p *=* *0.42
	PROT 2	531.89 ± 153.83 nm	40.73 ± 108.94 nm	*t*_(3)_ = 0.75	*p *=* *0.25
	PROT 3	548.70 ± 142.36 nm	57.54 ± 99.79 nm	*t*_(3)_ = 1.15	*p *=* *0.17
	PROT 4	461.80 ± 141.97 nm	−29.36 ± 47.56 nm	*t*_(3)_ = −1.23	*p *=* *0.15

The average initial offsets after mechanical overstimulation were comparable for all of the efference stimulus paradigms (Fig. 8*A*). The hair bundles were subdivided and further analyzed. The mean initial offsets of the three subcategories are shown in the $〈X_{o}〉$ column. With respect to protocol 0, the differences in initial offsets and the results of one-tailed paired *t* tests are listed. Differences were considered significant if *p *<* *0.05 and are indicated by asterisks. Hence, there were no statistically significant differences in the initial offsets, for any of the subgroups of hair cells and under any of the efference paradigms.

Furthermore, we explored whether efferent modulation during the postdeflection recovery period (protocol 4) had an impact on the timescales of baseline recovery with respect to the timescales observed under the control condition (protocol 0). It has been shown that the time course of the slow component of recovery is well described by the two-exponent function in Equation 1 (Kao et al., 2013). In order to quantify the timescale of recovery, we extracted the characteristic time constants, *τ*_1_ and *τ*_2_ ( $\tau_{2}>\tau_{1}$) by fitting the empirical baselines to Equation 1.

The time constants of the recovery extracted from recordings of 22 cells across nine sacculi, with simultaneous efferent actuation, are shown in the histogram of Figure 8*B*. The averages of *τ*_1_ and *τ*_2_ are *τ*_1_ = 55 ± 26 and *τ*_2_ = 590 ± 314 ms, respectively. The calculated baseline time constants for the same 22 bundles under the efference off condition are illustrated in the histogram of Figure 8*C*, and their respective averages are *τ*_1_ = 52 ± 20 and *τ*_2_ = 710 ± 588 ms. All baselines were uniquely fitted with *R*^2^ > 0.95. With respect to the control condition (efference off), the means of the paired differences are Δ(*τ*_1_) = 3 ± 15 ms (one-tailed paired *t* test, *t*_(21)_ = 0.87, *p *=* *0.20) and Δ(*τ*_2_) = – 120 ± 457 ms (one-tailed paired *t* test, *t*_(21)_ = −1.23, *p *=* *0.12). Thus, efferent activity did not induce a statistically significant difference in either *τ*_1_ or *τ*_2_. This result, in addition to the similarity between the distributions illustrated in Figure 8*B*,*C*, indicate that efference did not substantially alter a hair bundle’s overall resumption of its steady state position. This result is accordant with our finding that efferent modulation does not manifest statistically significant differences in the hair bundle’s induced positional offset.

## Discussion

The present work assesses the effects of efferent actuation on a hair bundle’s recovery dynamics subsequent to mechanical overstimulation. We approached this task by examining the time evolution of a hair bundle’s oscillatory profile as it recovers from a large-amplitude mechanical deflection while applying different modes of efferent stimulation.

### Mechanical overstimulation induces a transition in the dynamic state of the hair bundle

The hair bundle exhibits a complex temporal profile as it relaxes back to its equilibrium position following the cessation of mechanical overstimulation. While loud sounds would naturally occur at specific frequencies, our prior studies of overstimulation showed that the effect on saccular hair bundles was not dependent on the frequency of the stimulus, but rather on the duration of the applied signal (Kao et al., 2013). For simplicity, we hence focused on steady state deflections of the bundle and varied only the length of the stimulus. Our findings indicate that, with lengthening deflection durations, the hair cell’s burgeoning spontaneous oscillation frequency generally increases, while the amplitude and open probability typically decrease. The impact of prolonged deflection on hair bundle dynamics has not been fully explained and likely involves a combination of internal mechanisms. However, the strong dependency of the hair bundle’s incipient oscillation frequency, amplitude, and open probability on the duration of overstimulation point to a cumulative effect that seems to integrate over the presentation of mechanical stimulus.

When the hair bundle is deflected, the MET channels are held in a preferentially open state, allowing for the influx of cations, which are predominantly K^+^ with a fraction of the current carried by Ca^2+^ (Lumpkin and Hudspeth, 1995; Lumpkin et al., 1997; Hudspeth et al., 2000). In conjunction, Ca^2+^ pumps located in the stereovilli continuously extrude Ca^2+^ to restore a low internal resting concentration (Lumpkin and Hudspeth, 1998). As adaptation of the channel opening probability has been shown to be incomplete (Eatock, 2000), a deflection applied at a large amplitude is likely to lead to a prolonged ionic influx, which possibly overwhelms the extrusion pumps and leads to an accumulation of Ca^2+^ within the stereovilli. Studies involving manipulations of external Ca^2+^ concentration and blockers of extrusion pumps were consistent with this interpretation (Lumpkin and Hudspeth, 1998; Beurg et al., 2008; Kao et al., 2013). Another set of studies showed that depolarization of the hair cell soma strongly impacts the bundle oscillations in a manner implicating the modulation of internal Ca^2+^ (Meenderink et al., 2015; Khamesian and Neiman, 2017). The accumulation of Ca^2+^ in the stereovilli or the soma therefore constitutes a plausible mechanism for the impact of overstimulation. A direct effect of voltage on the bundle mechanics cannot be firmly excluded; however, voltage-mediated effects on bundle position have thus far been found to be transient (Assad et al., 1989) and hence unlikely to play a role in the slow recovery. Finally, the myosin motors which tune the optimal set point of the hair bundle are likely to be strongly offset by the deflection. The full biophysical effect, therefore, likely involves an interplay between depolarization of the soma, internal Ca^2+^ dynamics, and myosin motor offsets.

The formalism of nonlinear dynamics theory, however, provides a ready interpretation of this effect. A number of active nonlinear systems have been described using equations that exhibit two different dynamic states: a quiescent and an oscillatory state, with a critical point separating the two regimes. Spontaneous hair bundle oscillations have been shown to constitute active limit cycles, which are very well described by these simple models (Camalet et al., 2000; Nadrowski et al., 2004; Tinevez et al., 2007). Our results show that the application of a strong mechanical signal, which triggers a complex set of internal biophysical processes, ultimately modulates a control parameter that temporarily shifts the hair bundle into the quiescent state. Theoretical work on hair cell dynamics has long speculated that a control mechanism serves to tune its response (Choe et al., 1998; Camalet et al., 2000; Shlomovitz et al., 2013). The findings from this work and prior studies provide experimental evidence that active internal cellular processes self-tune the hair cell to the oscillatory state (Kao et al., 2013; Shlomovitz et al., 2013).

### The efferent system provides a biological mechanism for controlling the dynamic state of a hair cell bundle

Prior studies have extensively delved into the physiology of efferent neurons in mammalian species, with a particular focus on the medial olivocochlear (MOC) subset. These efferents synapse onto hair cells and stimulate the *α*9*α*10 nicotinic acetylcholine receptors (nAChR) by releasing ACh (Gómez-Casati et al., 2005; Ballestero et al., 2011; Guinan, 2011; Wedemeyer et al., 2018). Binding of ACh to the receptors then prompts a cascade of ion channel openings in the cell soma from which a complex spectrum of behaviors has been identified (Wersinger and Fuchs, 2011). Predominantly, however, efferent activation leads to hyperpolarization of the membrane potential (Art et al., 1984; Goutman et al., 2005). Prior studies in this field have specifically indicated that the influx of Ca^2+^ through the cholinergic receptors triggers the opening of SK2 channels and a consequent outflow of K^+^ (Blanchet et al., 1996; Oliver et al., 2000; Rohmann et al., 2015), which leads to an overall hyperpolarization of the soma (Fuchs and Murrow, 1992; Fuchs, 2002; Kong et al., 2008; Castellano-Muñoz et al., 2010).

Fully-developed hair cells in the American bullfrog’s saccular macula have been grouped into two classes, based on their morphology and electrophysiological properties (Chabbert, 1997; Rutherford and Roberts, 2009). The first type consists of flask-shaped cells that display large Ca^2+^-dependent K^+^ currents, and the second group contains cylindrical cells with large voltage-dependent Ca^2+^ currents. All hair cells in the bullfrog sacculus are highly innervated by efferent fibers, with the flask-shaped cells of the first class generally exhibiting more efferent contacts. Efferent fibers make direct contact with saccular hair cells, and there is an average of 10 efferent terminals per cell (Castellano-Muñoz et al., 2010). Electrophysiological measurements of hair cells from the American bullfrog sacculus likewise demonstrated the hyperpolarization of the hair cell soma as a result of efferent modulation (Castellano-Muñoz et al., 2010). Intensifying the strength of the efferent stimulation extended the hyperpolarization up to a saturation limit. The hyperpolarization of the hair cell soma in response to efferent modulation appears to be a consistent phenomenon observed in auditory and vestibular hair cells across a number of species studied (Art et al., 1984; Goutman et al., 2005; Wersinger and Fuchs, 2011). Furthermore, the results of our previous work, which linked efferent activity and active hair bundle motility, were in agreement with the hyperpolarization of the membrane potential (Lin and Bozovic, 2020). Specifically, characteristic aspects of a hair bundle’s spontaneous oscillation profile (i.e., frequency, amplitude, open probability) transformed in a manner that concurred with electrophysiologically hyperpolarizing the hair cell. Moreover, the changes in oscillation shape were found to depend on the level of efferent modulation in a way that corresponds with increasing hyperpolarization of the cell soma. Therefore, given the similarity between many of the observed effects of efferent actuation across different systems, we propose that the saccular epithelium provides a useful experimental model for examining the impact of efference on the hair bundle response.

Two characteristic features resulting from a high-amplitude mechanical deflection of the hair bundle are a transient induced offset in the bundle position and a temporary crossover from the oscillatory regime to the quiescent state. As it had been previously proposed that an imposed mechanical offset could control a hair bundle’s dynamic state, a plausible hypothesis that explains how stimulating the efferent neurons results in our observed findings is that efference modifies the net offset accrued from overstimulation. However, under conditions in which the efferents were active during the bundle’s relaxation from overstimulation, we regularly observed hair bundles in an oscillatory state while still at a significant mechanical deflection. Thus, actuating the efferent neurons is capable of producing an immediate transition from the quiescent state back to the oscillatory regime even at a large positional offset. Overall, examination of the induced offsets showed that efferent actuation had no impact under any of the efference paradigms, thereby eliminating the hypothesis that efferent control is mediated by modulation of the mechanical steady state.

A likely pathway by which the efferent system exerts its effect on hair cells is through modulation of the somatic potential, which in turn affects Ca^2+^ feedback processes within the sterovilli. Specifically, Ca^2+^ influx has been seen to affect a hair bundle’s dynamics at multiple timescales (Eatock, 2000; Hudspeth et al., 2000; Fettiplace, 2017), from Ca^2+^-mediated channel reclosure and control of adaptation motors (Howard and Hudspeth, 1987, 1988; Assad and Corey, 1992; Walker and Hudspeth, 1996; Gillespie and Corey, 1997; Ricci et al., 1998) to modulation of an internal gating spring stiffness (Marquis and Hudspeth, 1997; Martin et al., 2000; Ricci et al., 2002; Beurg et al., 2008; Roongthumskul et al., 2011). For the hair cells of the American bullfrog sacculus, channel reclosure occurs on the order of a few milliseconds, myosin motor adaptation takes place in ∼20 ms, and the timescale for modulation of an internal gating spring is ∼100 ms (LeMasurier and Gillespie, 2005; Roongthumskul et al., 2011). However, our results indicate that the baseline of recovery is unaffected by efferent activity for either of the experimentally determined timescales. This finding eliminates the possibility that regulation of myosin motor activity is the dominant outcome, as that would be reflected in the time constants characterizing the recovery. Hence, some sort of interplay between the somatic potential and Ca^2+^ effects on ion channels and gating spring mechanics still remains a valid hypothetical explanation.

Actuation of the efferent neurons before the mechanical deflection did not exert a measurable effect. This indicates that any shift that might have been induced in the internal dynamic state of the cell is transient and does not affect the subsequent recovery. Similarly, there was no observed response when the efferents were only activated simultaneously with the mechanical stimulus. This shows that the consequences of the mechanical overstimulation were dominant, which is consistent with the lack of effect on the accrued positional offset. Lastly, the almost instantaneous response to efferent modulation during the postdeflection recovery suggests that the efference-generated variation of internal elements overrides the tensing of the tip links by the mechanical deflection.

We also note that, while activation of nAChR receptors seems to be a requisite component of the efferent effect on hair bundle dynamics (Lin and Bozovic, 2020), we cannot eliminate the possible role of other neuromodulators reported to constitute a portion of the efferent mechanism (Kitcher et al., 2022). Parsing the confluence of the different cellular processes comprising the efferent feedback system represents a future direction for both theoretical and experimental studies.

In summary, the findings reported in this study provide evidence that the efferent neurons may serve as the biological feedback mechanism that controls the dynamic state of the hair cell. Specifically, our results demonstrate that the concurrent actuation of the efferent neurons with a hair cell’s postoverstimulation recovery can eliminate the usual suppression of oscillations, even at large positional offsets. Furthermore, this return to the oscillatory state appears to be decoupled from the slow recovery of steady state position. In conjunction with our prior findings, which demonstrated that efferent modulation can reversibly reduce a hair cell’s sensitivity to weak mechanical signals (Lin and Bozovic, 2020), the observation that efferent activity can eliminate the transition to quiescence induced by strong mechanical forcing gives an indication of how the efferent architecture might be enhancing the robustness of the hair cell and thus protecting it from damage.

## Synthesis

Reviewing Editor: David Schoppik, New York University - Langone Medical Center Decisions are customarily a result of the Reviewing Editor and the peer reviewers coming together and discussing their recommendations until a consensus is reached. When revisions are invited, a fact-based synthesis statement explaining their decision and outlining what is needed to prepare a revision will be listed below. The following reviewer(s) agreed to reveal their identity: NONE. Note: If this manuscript was transferred from JNeurosci and a decision was made to accept the manuscript without peer review, a brief statement to this effect will instead be what is listed below.

Based on the responses to reviewers I think the revised manuscript successfully addresses the prior concerns and is the stronger for it.
